# Immunocytochemical study of TOP2A and Ki-67 in cervical smears from women under routine gynecological care

**DOI:** 10.1186/s12929-016-0258-z

**Published:** 2016-05-12

**Authors:** Adrya Lúcia Peres, Keilla Maria Paz e Silva, Rosângela Ferreira Frade de Araújo, José Luiz de Lima Filho, Mário Ribeiro de Melo Júnior, Danyelly Bruneska Gondim Martins, Nicodemos Teles de Pontes Filho

**Affiliations:** Laboratory of Immunopathology Keizo Asami (LIKA), Federal University of Pernambuco (UFPE), Recife, Brazil; Molecular Prospection and Bioinformatics Group - Laboratory of Immunopathology Keizo Asami (LIKA), Federal University of Pernambuco (UFPE), Recife, Brazil; Department of Biochemistry, Biological Science Centre, Federal University of Pernambuco (UFPE), Recife, Brazil; Faculdade ASCES/Associação Caruaruense de Ensino Superior, Caruaru – PE, Brazil; Department of Pathology, Healthy Science Centre, Federal University of Pernambuco (UFPE), Recife, Brazil

**Keywords:** Cervical cytopathology, Human papillomavirus, Ki-67 antigen, DNA Topoisomerases type IIa, Immunocytochemistry

## Abstract

**Background:**

Cervical cancer is one of the most common female cancers and is caused by human papillomavirus (HPV). Viral infection leads to cell cycle deregulation by inactivating p53 and retinoblastoma protein by viral oncoproteins E6 and E7, respectively. Then, nuclear proteins such as DNA topoisomerase type IIa (TOP2A) and Ki-67 show increased expression because of increased cell division. These molecules are used as biomarkers for immunohistochemistry analysis of cervical tissue.

**Methods:**

In this cross-sectional study, we recruited 110 women receiving regular gynecological surveillance at public health centers in Olinda – PE, Brazil. Cervicovaginal cells were collected to determine the presence of cytological abnormalities and HPV infection. Pap smear slides were used to evaluate the expression of TOP2A and Ki-67 using immunocytochemistry techniques.

**Results:**

Of the 110 women, 75.4 % showed HPV-DNA^+^ infection (83/110) and 29.1 % showed cellular abnormalities (32/110). Two atypical cells of undetermined significance, one low-grade squamous intraepithelial lesion, and one high-grade squamous intraepithelial lesion samples showed no HPV-DNA. TOP2A was positive in 71.9 % of samples, while Ki-67 was positive in 81.2 %. Immunocytochemistry results were positive in 4 of 5 atypical cells of undetermined significance samples. In HPV-DNA^+^ samples with cytological abnormalities, immunocytochemistry results were positive 96.4 % of samples (*p* < 0.0001; odds ratio = 28.0). Among the samples infected with HR-HPV, TOP2A^+^ was effective in 71 % samples, while and Ki-67^+^ was 77.4 %. Ki-67 and TOP2A were positive for all samples infected with HPV6, HPV11, and HPV18. Ki-67 was also positive for all HPV16 samples, except for one negative sample in cytopathology analysis.

**Conclusions:**

TOP2A and Ki-67 antibodies may be used in combination for cervical cancer screening in immunocytochemistry assays.

## Background

Worldwide, cervical cancer is the third most common female cancer. In 2012, 527,624 cases were reported. This number is expected to increase by 6.23 % in 2015, reaching to 560,505 cases [1, 2]. Cervical cancer development is associated with persistent human papillomavirus (HPV) infection. Among more than 100 HPV genotypes, HPV16 and HPV18 alone are responsible for 73 % of cervical cancer cases [3, 4].

The viral oncoproteins E6, E7, and E5 deregulate the cell cycle and structure [5, 6], leading to cellular proliferation and morphological abnormalities. Therefore, molecules related to cell cycle regulation [p53, p21, Bcl-2-associated X protein, p27, retinoblastoma protein, p16 INK4a, minichromosome maintenance protein 2 (MCM2), topoisomerases type IIa (TOP2A), cyclin D1, cyclin A, and cyclin E] and cell proliferation (proliferating cell nuclear antigen, Ki-67, and c-myc) are used as biomarkers for cervical cancer diagnosis [6].

TOP2A is responsible for enzymatic uncoupling during the replication of DNA strands, playing a significant role in S-phase regulation. TOP2A is overexpressed during the progression from cervical intraepithelial neoplasia 2 (CIN2)-CIN3 to cervical cancer, and thus it is considered to be a late marker of cervical carcinogenesis [7, 8]. The combination of TOP2A and MCM2 in the commercial assay ProExC™ is used for immunohistochemistry analysis in cervical cancer samples.

Ki-67 is a nuclear and nucleolar protein expressed during all phases of the cell cycle, except for the G0 phase [8]. Ki-67 expression is typically confined to the basal and parabasal layers of the normal cervical squamous epithelium. However, in dysplasia and carcinoma, expression extends above one-third of the epithelium. Therefore, Ki-67 is a reliable marker for cell cycle deregulation caused by HPV infection and is related to the risk of progression from precursor lesions to carcinoma [9, 10].

The aim of this study was to determine the feasibility of using immunocytochemistry for TOP2A and Ki-67 on cytopathology slides to improve the accuracy of cervical cancer screening.

## Methods

### Study design and case selection

This cross-sectional study included women under surveillance at public health centers in Brazil. Samples consisted of 110 patients aged 13–71 years from 3 familial health units located in Olinda-PE. During regular gynecological inspection, all patients underwent Pap testing and HPV-DNA investigation. Exclusion criteria were: HIV infection, pregnancy and post-partum, history of transplant, history of partial or complete uterus removal, and immunocompromised patients. This study was approved by institutional ethical committee Health Sciences Center Federal University of Pernambuco, Brazil (Code No.275/08) and conducted according to the guidelines of the Declaration of Helsinki (1964).

### Cervical samples

Cervical samples were harvested from the ectocervix and endocervix using a spatula and cytobrush (Kolplast, São Paulo, Brazil), respectively. Collected cells were transferred to a glass slide, identified, and then fixed in absolute ethanol (Vetec, Rio de Janeiro, Brazil; Sigma-Aldrich, St. Louis, MO, USA) until sample preparation for cytopathology and immunocytochemistry analysis at Pathology Sector – LIKA/UFPE. The residual cells in the cytobrush were stored in phosphate-buffered saline (PBS), pH 7.0 (Life Technologies, USA) at −20 °C before HPV identification and genotyping at Molecular Prospection Sector – LIKA/UFPE. For analysis, patients were divided into four groups according to Pap test and HPV-DNA identification: (1) women with cervical abnormalities and HPV-DNA positive; (2) women with cervical abnormalities and HPV-negative; (3) women with normal cytology and HPV-DNA positive; and (4) women with normal cytology and HPV-negative (control group).

### Cytological analysis

All samples underwent Papanicolaou staining [11] and were categorized according to the 2001 Bethesda System nomenclature for cervical cytology. Four categories were used, as follows: negative for intraepithelial lesion or malignancy (NILM), atypical squamous cells of undetermined significance (ASC-US), low-grade squamous intraepithelial lesion (LSIL), and high-grade squamous intraepithelial lesion (HSIL) [12]. Two independent cytopathologists (A.L.P. and N.T.P.F) assessed all slides in a double-blinded manner and contradictory findings were resolved through discussion.

### HPV-DNA identification

Total DNA extraction was performed using the Wizard Genomic DNA Purification Kit (Promega, Madison, WI, USA) following the manufacturer’s instructions. Eluted DNA was stored at −20 °C until HPV-DNA identification. Conventional PCR was performed in a Verity® Thermal Cycler (Thermo Fischer Scientific, Waltham, MA, USA) using two consensus primers: GP5+/GP6+ [13] or MY09/MY11 [14] purchased from Integrated DNA Technologies (Coralville, IA, USA). β-Actin was used as a housekeeping gene (Fw: 5′-AGC GGG AAA TCG TGC GTG-3′ and Rv: 5′-GGT GAT GAC CTG GCC GTC-3′) to ensure the quality of DNA extracted. The reaction mix was prepared with 1 μL DNA (80–100 ng), 1 μM of each primer, and 12.5 μL Go Taq Green MasterMix (Promega) in a final volume of 25 μL. The PCR was performed as follows: (i) initial denaturation at 94 °C for 2 min; (ii) 34 cycles of denaturation at 95 °C for 1 min, (iii) annealing at 45 °C (for GP5+/6+) or 55 °C (for MY09/11) or 60 °C (for β-actin) for 1 min; and (iv) extension at 72 °C for 1 min. A final extension was performed at 72 °C for 5 min. A plasmid containing the HPV16 complete genome was used as a positive control. Ultrapure water was used as a negative control. Amplicons were observed on a 1 % agarose gel stained with ethidium bromide (0.5 mg/mL final concentration). All samples were analyzed twice for each primer pair in independent experiments. HPV-positive results were assumed for samples with at least two positive results in HPV amplification.

### HPV-DNA genotyping

Cervical samples assumed as positive for HPV-DNA or presenting cytological abnormalities were evaluated using the PapilloCheck® Test Kit (Greiner Bio-One GmbH, Frickenhausen, Germany), according to the manufacturer’s instructions. This chip-based assay identifies 18 HPV genotypes classified as high-risk (HR) or probable HR-HPV (16, 18, 31, 33, 35, 39, 45, 51, 52, 53, 56, 58, 59, 66, 68, 70, 73, 82) and 6 low-risk (LR) HPV (HPV6, 11, 40, 42, 43, 44/55). The test cannot distinguish between HPV-55 and HPV-44 because of a cross-reaction, and the manufacturer instructions do not differentiate HR-HPV from probable HR-HPV. Slide scanning was performed using a Greiner Bio-One CheckScanner™. Data analysis was performed using CheckReport™ Software. Since PapilloCheck® requires highly pure and concentrated DNA (400–800 ng input), all samples that did not fulfill the criteria were excluded from the study.

### Immunocytochemistry procedures

**Fig. 1:**
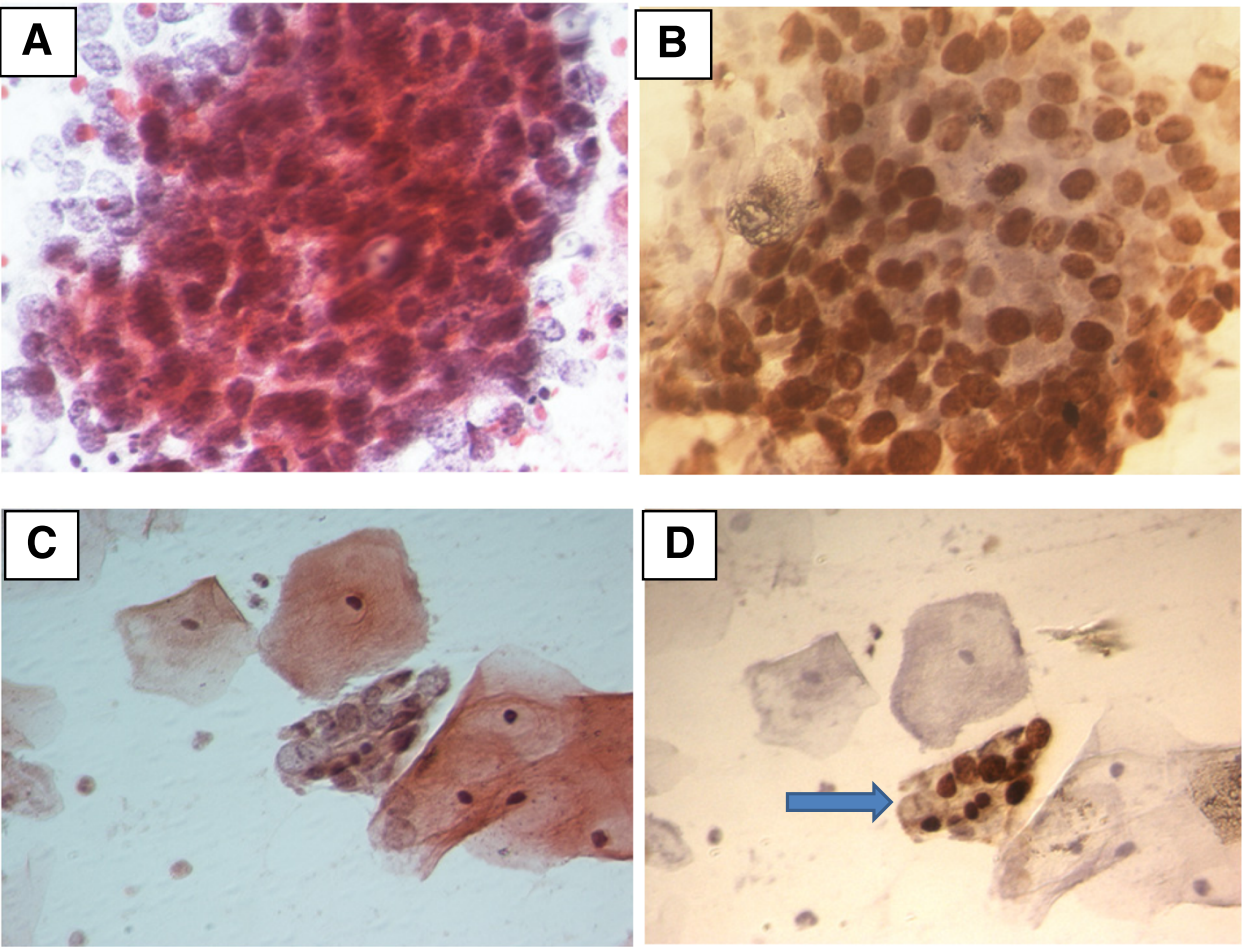
**a** Papanicolaou staining of cervical cells showing syncytial arrangement with dyskaryosis characterizing HSIL (magnification: 400×). **b** TOP2A-positive immunocytochemistry of cervical cells at similar syncytial arrangement (magnification: 400×). **c** Papanicolaou staining of cervical cells showing syncytial arrangement with dyskaryosis, (magnification: 400×). **d** Ki-67-positive immunocytochemistry of cervical cells with similar syncytial arrangement (400×)

Immunocytochemistry (ICC) was performed on the Pap smear slides by removing coverslips in xylene and rehydrating the samples in decreasing concentration of ethyl alcohol [15]. To test two antibodies (anti-TOP2A and anti-Ki-67) on the same slide, a hydrophobic barrier was introduced using the Dako Pen S2002 (Dako, Carpinteria, CA, USA) to prevent mixing of the antibody solutions. The design of the separation line occurred after careful observation of the slide to ensure the presence of cells with morphological abnormalities on both sides of the barrier. Counterstain using Harry’s hematoxylin was performed before slide assembly. Immunostaining was performed with antibodies against Ki-67 (Clone MIB-1, Dako) and TOP2A (Clone Ki-S1, Dako) at 1:50 and 1:100 dilutions, respectively. Both antibody solutions were prepared in PBS, pH 7.2, containing 1 % bovine serum albumin (Thermo Fischer Scientific). Starr Trek Universal HRP Detection System (BIOCARE MEDICAL, Concord, CA, USA) was used according to manufacturer’s instructions. Slides were evaluated using an Olympus microscope (Tokyo, Japan) at 400× magnification. Two slides well-known to be immunoreactive for Ki-67 and TOP2A were used as positive controls in each batch: (1) a slide containing a tissue section from cervical cancer biopsy and (2) a slide containing cytological samples from invasive squamous cell carcinoma. PBS, pH 7.2, was used as a negative control. Slides were assumed to be positive for TOP2A or Ki-67 when at least one cell showed a dark brown nucleus (Fig. 1a–d). Immunocytochemistry results were categorized into four groups: Ki-67^+^; TOP2A^+^; Ki-67^+^/TOP2A^+^ (positive for both markers); and Ki-67^+^ and/or TOP2A^+^ (positive for at least one marker on the same slide), designated as ICC^+^. Two independent cytopathologists (A.L.P. and N.T.P.F) assessed all slides in a double-blinded manner; contradictory findings resolved through discussion.

### Statistical analysis

Statistical analysis was performed using Prism software v6.0 (GraphPad, Inc., La Jolla, CA, USA). The association between groups according to cytopathology, immunocytochemistry, and PCR results were performed by Chi-square test or Fischer exact test with 95 % confidence intervals (95 % CI) and odds ratio (OR). Statistics were significant when *p* < 0.05. The concordance between Ki-67 and TOP2A results were analyzed using Kappa statistics (κ) with 95 % CI in BioEstat software (Version 5.3).

## Results

### Sample characterization

Among the 110 patients, the median age was 30 years, 77.8 % declared household incomes up to two minimum wages, and 95.4 % had no more than a high school education level (Table 1). Most women were married (53.7 %), reported the first sexual intercourse after 16 years old (59.4 %), and had fewer than three sexual partners (51.4 %). Multiparous women represented 56.4 % of the patients, varying from 2 to 11 children each. Abortion was reported by 33.6 % of the patients, including two nulliparous women. Only 1.8 % of the patients reported the use of oral contraceptive methods. A total of 28.2 % of women reported tubal ligation, a factor for HPV infection in this group (*p* = 0.0013; OR = 4.779). The youngest patient to report tubal ligation was 26 years, she used no other contraceptive method, had her first pregnancy at 19 years, and had 5 children. Smoking habits and alcohol consumption were reported by 34.5 and 54.5 % of patients, respectively.

**Table 1:** Socio-demographic profile and clinical status of 110 patients enrolled in the study

		HPV infection		
Characteristics	*N*	*N*	(%)	*p* value
Total samples	110	83	75.4	0.3907
Age, years				
< 16	7	6	85.7	
16–30	49	34	69.4	
> 30	54	43	79.6	
Household income, minimum wage (R$)^a^				0.5369
< 2	84	65	77.4	
2–5	25	17	68.0	
> 5	1	1	100.0	
Educational level				0.4321
High school or less	105	79	75.2	
Incomplete college	3	3	100.0	
College graduate	2	1	50.0	
Matrimonial status^b^				0.4050
Single	38	27	71.0	
Married	58	46	79.3	
Divorced	8	5	62.5	
Widow	4	4	100.0	
Age of first sexual intercourse, years^c^				0.2414
< 16	43	36	83.7	
≥ 16	63	46	73.0	
Sexual partners^d^				0.3956
< 3	54	39	72.2	
3–7	46	34	73.9	
> 7	5	5	100.0	
Parity				0.6964
Nulliparous	27	19	70.4	
Primiparous	21	17	80.9	
Multiparous (2–11)	62	47	75.8	
Abortion^e^				0.6082
Yes (1–5)	37	27	72.9	
No	48	38	79.2	
Oral contraceptive				1.00
Yes	2	2	100.0	
No	108	81	75	
Tubal ligation				0.0013
Yes	31	24	77.4	
No	79	33	41.8	
Smoking habit				0.6439
Yes	38	30	78.9	
No	72	53	73.6	
Alcohol consumption				0.0754
Yes	60	41	68.3	
No	50	42	84.0	

^a^Minimum wage = R$ 622.00; ^b^2 patients did not answer the question; ^c^4 patients did not answer; ^d^5 patients did not answer; ^e^excluding 25 patients that never were pregnant

Of the 110 women, 75.4 % showed HPV-DNA^+^ infection (83/110) and 29.1 % showed cellular abnormalities (32/110). Twenty-eight samples were positive for both tests, cytopathology, and HPV-DNA (87.5 %). Two ASC-US, one LSIL, and one HSIL sample showed no HPV-DNA (Table 2).

**Table 2:** Immunocytochemistry profile of TOP2A and Ki-67 antibodies related to cytopathology classification of cervical smears

Cytopathology	*N*	HPV^+^ (%)	Immunocytochemistry			
			TOP2A^+^ (%)	Ki-67^+^ (%)	TOP2A^+^/Ki-67^+^ (%)	ICC^+^ (%)
Positive	32*^,^**^,^***^,^****	28 (87.5)	23* (71.9)	26** (81.2)	19*** (59.4)	30**** (93.7)
ASC-US	5	3 (60)	2 (40)	3 (60)	1 (20)	4 (80)
LSIL	19	18 (94.7)	13 (68)	16 (84)	11 (57.9)	18 (94.7)
HSIL	8	7 (87.5)	8 (100)	7 (87.5)	7 (87.5)	8 (100)
NILM	78	55 (70.5)	28 (36)	21 (27)	18 (23)	31 (39.7)
Total	110	83	51	47	37	61

*p < 0.0007 (OR = 4.563; 95 % CI: 1.857–11.21); **p < 0.0001 (OR = 11.76; 95 % CI: 4.245–32.59); ***p < 0.0007 (OR = 4.872; 95 % CI: 2.019–11.75); ****p < 0.0001 (OR = 22.74; 95 % CI 5.065–102.1)

### Immunocytochemistry analysis

In samples with cytological abnormalities, TOP2A was positive in 71.9 % of the samples, while Ki-67 was positive in 81.2 % of the samples. Comparison of the two markers showed that TOP2A was more efficient for HSIL, as expected for a prognosis cancer biomarker. Ki-67 was more effective for detecting ASC-US and LSIL, as it indicates cellular proliferation. The positive rate of TOP2A and Ki-67 increases when cervical lesion severity increases. The concordance between TOP2A and Ki-67 results was 78.2 % (κ = 0.56; moderate agreement; *p* < 0.0001).

When evaluated as a single test, the positive rate for ICC^+^ was 93.7 % in the group with cervical abnormalities. The ICC^+^ test showed OR = 22.74, which was higher than those of Ki-67^+^ (OR = 11.76) and TOP2A (OR = 4.56). The concordance between the cytopathology and ICC test was 70 % (κ = 0.43; *p* < 0.0001).

The samples categorized as NILM corresponded to 70.9 % (78/110). Among these samples, 70.5 % (55/78) were HPV-DNA^+^. TOP2A^+^ and Ki-67^+^ were observed in 36 and 27 % of samples, respectively. ICC^+^ results were observed in 39.7 % of samples (31/78). Twenty-seven samples (49.1 %) were HPV-DNA^+^ and ICC-negative. Only four samples (7.3 %) were HPV-negative and ICC^+^.

Table 3 shows TOP2A and Ki-67 staining according to HPV infection. In HPV-DNA^+^ samples with cytological abnormalities, the ICC^+^ result was 96.4 % (*p* < 0.0001; OR = 28.0). In the HPV-negative group, the overall ICC^+^ rate was 33.3 %, identifying 3 of 4 HPV-negative samples. The overall ICC^+^ result in HPV-DNA^+^ samples was 64 %, with similar positivity between both markers.

**Table 3:** Immunocytochemistry analysis related to HPV-DNA in 110 cervical samples

Human papillomavirus	*N*	Ki-67^+^			TOP2A^+^			Ki-67^+^/TOP2A^+^			ICC^+^ (%)		
		*N* (%)	*p*	OR (95 % IC)	*N* (%)	*p*	OR (95 % IC)	*N* (%)	*p*	OR (95 % IC)	*N* (%)	*p*	OR (95 % IC)
HPV^+^	83	45 (54.2)	0.0003	6.809 (2.16–21.43)	45 (54.2)	0.0142	3.383 (1.29–8.86)	36 (43.4)	0.0004	9.574 (2.12–43.11)	54 (65.1)	0.0065	3.724 (1.48–9.33)
With cytological abnormalities	28	26 (92.8)	0.0001	24.63 (5.26–115.2)	20 (71.4)	0.0357	3.00 (1.12–7.96)	19 (67.8)	0.0021	4.791 (1.77–12.55)	27 (96.4)	0.0001	28.00 (3.55–220.8)
W/o cytological abnormalities	55	19 (34.4)			25 (45.4)			17 (30.9)			27 (49.1)		
HPV^−^	27	4 (14.8)			7 (25.9)			2 (7.4)			9 (33.3)		
With cytological abnormalities	4	0 (−)	0.4286	–	3 (75)	1.00	–	0 (−)	1.00	–	3 (75)	1.00	–
W/o cytological abnormalities	23	2 (8.7)			3 (13.0)			1 (4.3)			4 (17.4)		

Samples infected with HPV-DNA or showing cytological abnormalities were selected for genotyping. A total of 83 samples were considered for genotyping, but seven samples were excluded because of low quality, including three ASC-US and one LSIL. Therefore, 76 samples were evaluated for HPV genotypes, including 28 samples with cytological abnormalities and 74 HPV-DNA^+^ samples.

HPV genotypes were identified in 34 samples (43.6 %), of which 31 (91.2 %) were infected with the HR-HPV genotypes. Nine patients were infected with HPV16 and three patients were infected with HPV18 (Table 4). Thirteen samples showed multiple infections including up to 3 HPV genotypes. One patient was infected with HPV16 and HPV18. Only one sample (LSIL) was infected exclusively with LR-HPV genotypes (HPV6 and HPV11).

**Table 4:** Frequency of HPV genotypes in 34 positive samples, according to cytopathology and immunocytochemistry tests

HPV genotype	No. (%)	Cytopathology			Immunocytochemistry			
		ASC-US	LSIL	HSIL	Ki-67^+^	TOP2A^+^	Ki-67^+^/TOP2A^+^	ICC^+^
LR-HPV genotypes								
6	2 (5.9)	–	2	–	2	2	2	2
11	2 (5.9)	–	1	–	2	2	2	2
42	2 (5.9)	–	1	–	2	2	2	2
43	1 (2.9)	–	–	–	–	–	–	–
HR-HPV genotypes								
16	9 (26.5)	–	5	2	8	6	6	8
18	3 (8.8)	–	–	2	3	3	3	3
31	1 (2.9)	–	–	1	1	1	1	1
35	2 (5.9)	–	1	–	2	2	2	2
39	1 (2.9)	–	–	–	–	–	–	–
45	1 (2.9)	–	1	–	1	–	–	1
51	4 (11.8)	–	1	–	2	3	2	3
52	4 (11.8)	1	1	1	4	3	3	4
53	4 (11.8)	–	1	–	2	2	2	2
56	6 (17.4)	–	3	1	5	4	3	6
59	1 (2.9)	–	–	–	1	1	1	1
66	3 (8.8)	1	1	–	3	1	1	3
68	2 (5.9)	–	–	1	1	2	1	2
70	1 (2.9)	–	–	–	–	–	–	–
73	1 (2.9)	–	1	–	1	–	–	1
82	3 (8.8)	1	2	–	3	2	2	3

Among the samples infected with HR-HPV, we found 71 % TOP2A^+^ samples and 77.4 % Ki-67^+^ samples. Ki-67 and TOP2A were positive for all samples infected with HPV6, HPV11, and HPV18. Ki-67 was also positive for all HPV16 samples, except for one negative sample in cytopathology analysis. Twenty-seven samples (37.2 %) showed negative results for the Papillocheck test, including one HSIL (ICC^+^), two LSIL samples, and one ASC-US (HPV-DNA^+^ and ICC^+^). Genotyping tests failed in 15 samples, including two LSIL (ICC^+^). Of the 37 NILM samples evaluated, 13 samples were infected with HR-HPV and one sample was infected with LR-HPV.

## Discussion

Our findings showed a high positive rate for the ICC^+^ test in samples with cytological abnormalities. TOP2A was more effective for HSIL samples, while Ki-67 was more effective for ASC-US and LSIL. A combination of markers is necessary for cervical cancer screening systems when the concordance between markers shows only moderate agreement. TOP2A and Ki-67 were also useful for identifying samples infected with HR-HPV, an important result the potential of these markers for commercial application.

HPV-DNA tests exhibit high sensitivity but low specificity and generally cannot distinguish clinically relevant lesions from transient infections [16]. Thus, complementary biomarkers are needed to allow for such discrimination [17]. ICC may improve the management of cervical intraepithelial lesions following identification of protein expression deregulation [18, 19]. Careful interpretation of immunostaining results and morphological characteristics in conventional Pap smears can be a very useful as laboratory approach. This approach is also simple, reliable, and easily applicable in diagnostic routines [6, 19].

Application of the Ki-67 marker has been investigated in cervical cytology using classic Pap smears as a diagnostic adjunct for reducing the need for tissue biopsies [18, 20–22]. Ki-67 immunocytochemistry shows good accuracy for HSIL diagnosis and is used as an adjunct to cytology [6, 23]. In the present study, Ki-67 was positive in 81.2 % samples with cellular abnormalities, which is comparable to the results of previous studies [19, 24]. Since Ki-67 is highly expressed epithelial cells during proliferation, its positive staining can indicate an active HPV infection [8, 25]. Studies of cellular biomarkers in this field have described Ki-67 as a marker in cervical ASC-US. Ki-67 tests may help to reduce the rate of ASC-US, leading to differentiation of dysplasia from mimics such as atypical metaplasia or atrophy in diagnostically problematic cases [9, 26], although it is relatively nonspecific for detecting cervical squamous dysplasia [23, 27].

TOP2A and Ki-67 were previously tested together for immunohistochemistry in carcinoma and high-grade cervical neoplasia (CIN2/3), revealing no relationship between stained nuclei counting and survival [28]. However, another report showed that only TOP2A matched to all HSIL samples, with better results than for Ki-67 [27, 29], which agree with our ICC results. Studies also observed Ki-67 and TOP2A in the vulvar epithelium, showing good correlation for characterizing benign, intraepithelial, and invasive neoplastic epithelial changes [30].

In our study, using both markers enabled identification of cellular abnormalities in four of five ASC-US samples, corroborating the results of previous studies focused on atypical squamous cell cytology [5, 8, 27, 31]. We also identified structural modifications in samples with cytological abnormalities without HPV-DNA identification. This observation is in agreement with studies using SurePath preparation, in which ICC with p16 and Ki-67 could identify high protein expression even in HPV-negative ASC-US [10, 32] and ASC-H samples [5, 9]. HPV-DNA is normally positive in transient infections, accounting for the majority of infected cases. However, TOP2A staining as a maker of cell-cycle deregulation and Ki-67 for uncontrolled proliferation are expected to be positive only in dysplastic cells undergoing HPV-mediated transformation. A negative result for ICC in HPV^+^ samples indicates a low risk of cervical lesion development in these patients, although HPV is present.

The association of p16/Ki-67 staining increased sensitivity and specificity for identifying women at high-risk for developing high-grade precancerous disease in a group of HPV-positive patients with negative liquid-based Pap cytology [6, 33]. Singh et al. (2012) also applied dual staining for p16INK4a/Ki-67 ICC and showed increased specificity and remarkable sensitivity for diagnosing CIN2/3 or glandular lesions compared with PCR-based testing for HR-HPV [34]. Finally, CINtec® PLUS, a commercial assay for p16/Ki-67 determination, was strongly related to the presence of CIN2^+^ lesions and was associated with HR-HPV infection and an increasing grade of cervical lesions [7, 17]. In our study, TOP2A^+^ and Ki-67^+^ showed higher positive rates in samples infected with HR-HPV compared to in biopsied paraffin blocks analyzed for HPV16, 18, and 31 [35].

This is the first study investigating a combination of TOP2A and Ki-67 as markers for ICC in HR-HPV infection. The present study has some limitations, including the relatively small number of selected cases and the use of material collected during routine gynecological inspection of women under surveillance at public health centers in Brazil.

## Conclusions

TOP2A and Ki-67 may be a useful antibody combination for cervical cancer screening in health services currently using only conventional cytology. Further analyses are required before considering these markers for clinical follow-up in immunohistochemistry. Their performance must also be compared with those of commercial assays.

## Abbreviations

ASC-US: atypical squamous cells of undetermined significance
BSA: bovine serum albumin
CI: confidence intervals
CIN: neoplasia intraepithelial cervical
DNA: deoxyribonucleic acid
DNA-HPV: genome of HPV
HPV: human papillomavirus
HSIL: high-grade squamous intraepithelial lesion
ICC: immunocytochemistry
LSIL: low-grade squamous intraepithelial lesion
MCM2: minichromosome maintenance protein
NILM: negative for intraepithelial lesion or malignancy
OR: odds ratio
p16 INK4a: protein
PBS: phosphate-buffered saline
PCNA: proliferating cell nuclear antigen
PCR: polymerase chain reaction
pRb: protein retinoblastoma
ProExC™: kit antibody (anti-protein: TOP2A and MCM2 - minichromosome maintenance protein 2)
RR: relative risk
TOP2A: DNA topoisomerase II alpha
